# Extracorporeal membrane oxygenation as a bridge to lung transplant in the composite lung allocation score era: A single-center experience

**DOI:** 10.1016/j.xjon.2025.10.022

**Published:** 2025-11-04

**Authors:** Whitney D. Gannon, Enock Adjei, John W. Stokes, Todd W. Rice, Anil J. Trindade, Blaine M. Sklar, Christina A. Jelly, Aaron M. Williams, Konrad Hoetzenecker, Caitlin T. Demarest, Matthew Bacchetta

**Affiliations:** a Division of Allergy, Pulmonary and Critical Care Medicine, Vanderbilt University Medical Center, Nashville, Tenn; b Department of Thoracic Surgery, Vanderbilt University Medical Center, Nashville, Tenn; c Division of Cardiac Surgery, Vanderbilt University Medical Center, Nashville, Tenn; d Department of Anesthesiology, Vanderbilt University Medical Center, Nashville, Tenn; e Department of Biomedical Engineering, Vanderbilt University, Nashville, Tenn

**Figure:**
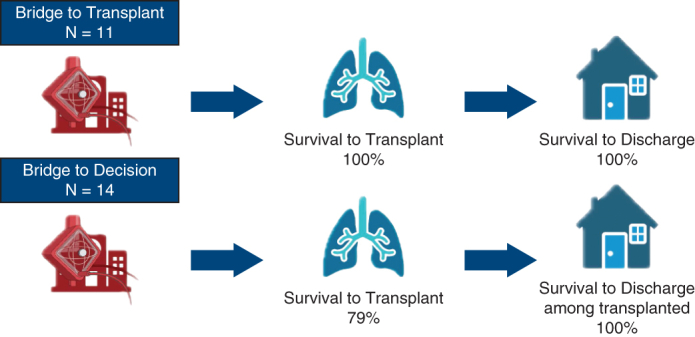
Survival in the ECMO-BTT and ECMO-BDT groups.

**Central Message**

Further work is critical to understand whether the introduction of CAS augments the potential benefit of ECMO as a bridge to lung transplant and allows provision of ECMO to higher-risk patients.

In selected patients with end-stage lung disease and severe respiratory failure, extracorporeal membrane oxygenation (ECMO) can facilitate awakening, rehabilitation, and avoidance of mechanical ventilation, thereby preserving lung transplant candidacy and optimizing outcomes after transplant.^1^ Although outcomes after lung transplant in patients who were bridged to transplant with ECMO are comparable to those patients not bridged with ECMO, survival rates to lung transplant on ECMO are variable.^1^ These varying outcomes may be influenced by prolonged wait times on ECMO,^1^ which are associated with removal from the waitlist due to ECMO-associated morbidity and higher pretransplant mortality.^2^

The composite lung allocation score (CAS) was introduced on March 9, 2023, to improve the efficiency of donor distribution and equitable access,^3^ and its use has reduced waitlist time and improved survival.^3^ The CAS incorporates priority points for the presence of ECMO, and recent data demonstrate a shorter waitlist time among patients receiving ECMO compared to the prior era.^4^ Therefore, it is possible that survival to transplant in patients receiving ECMO could be improved following implementation of the CAS. This might allow broader provision of ECMO to patients with higher-risk profiles, but data during the CAS era are lacking.

This is the first single-center cohort study at a high-volume lung transplant and ECMO center reporting clinical characteristics and outcomes of patients with end-stage lung disease who received pretransplant ECMO in the CAS era.

## Methods

We performed a retrospective cohort study examining data from all patients who received ECMO as either a bridge to lung transplant (ECMO-BTT; ie, the patient was actively listed for lung transplant at the time of cannulation) or bridge to the decision of whether to provide a lung transplant (ECMO-BTD; ie, the patient was not actively listed for lung transplant at the time of cannulation but was undergoing evaluation for lung transplant candidacy) at Vanderbilt University Medical Center between March 9, 2023, and July 20, 2025. The study was approved by the Vanderbilt University Institutional Review Board on August 1, 2025 (IRB no. 251167), with a waiver of patient consent.

We collected clinical characteristics and outcomes from the electronic medical record. Continuous variables are presented as median with interquartile range (IQR); categorical variables, as frequency and percentage. Outcomes included survival to transplant on ECMO, survival to hospital discharge and to 6 months after discharge, time on ECMO, and complications while receiving ECMO. We examined clinical characteristics and outcomes among the whole cohort and in the ECMO-BTT and ECMO-BTD groups. All analyses were performed using Stata 16.1 (StataCorp).

## Results

During the study period, 25 patients received either ECMO-BTT (n = 11) or ECMO-BTD (n = 14). Among the whole cohort, 20 patients (80%) had an underlying diagnosis of interstitial lung disease. The median patient age was 59 years (IQR, 54-65 years). Baseline patient and transplant characteristics were similar in the ECMO-BTT and ECMO-BTD groups (Table 1).

**Table 1:** Clinical characteristics and outcomes among 25 patients receiving pre–lung transplantation ECMO

Characteristic/outcome	Whole cohort (N = 25)	ECMO-BTT group (N = 11)	ECMO-BTD group (N = 14)
Age, y, median (IQR)	59 (54-65)	57.0 (54-60)	60 (55-65)
Female sex, n (%)	10 (40.0)	6 (54.6)	4 (28.6)
Body mass index, kg/m^2^, median (IQR)	27.9 (23.7-29.4)	26.5 (21.4-30.3)	27.9 (25.9-28.6)
Blood type, n (%)			
A	9 (36.0)	4 (36.4)	5 (35.7)
B	3 (12.0)	2 (18.2)	1 (7.1)
O	13 (52.0)	5 (45.5)	8 (57.1)
Ever smoker, n (%)	10 (40.0)	4 (36.4)	6 (42.9)
Underlying diagnosis, n (%)			
Interstitial lung disease	20 (80.0)	9 (81.8)	11 (78.6)
COPD	2 (8.0)	1 (9.1)	1 (7.1)
Cystic fibrosis/CLAD	2 (8.0)	1 (9.1)	1 (7.1)
Acute respiratory distress syndrome	1 (4.0)	0 (0.0)	1 (7.1)
History of prior lung transplant, n (%)	2 (8.3)	1 (9.1)	1 (7.1)
Immediately prior to ECMO			
Fraction of inspired oxygen, median (IQR)	1.0 (0.95-1.0)	1.0 (1.0-1.0)	1.0 (1.0-1.0)
Oxygen device, n (%)			
High-flow nasal cannula	22 (88.0)	11 (100.0)	11 (78.6)
Noninvasive positive-pressure ventilation	1 (4.0)	0 (0.0)	1 (7.1)
Invasive mechanical ventilation∗	2 (8.0)	0 (0.0)	2 (14.3)
Mean pulmonary artery pressure at cannulation, mm Hg, median (IQR)	32 (22-40)	33.5 (17.0-42.0)	29.0 (25.0-33.0)
Nonambulatory, n (%)	13 (52.0)	2 (18.2)	11 (78.6)
Initial configuration, n (%)			
Venoarterial venous	5 (20.0)	3 (27.3)	2 (14.3)
Venoarterial, sport model†	7 (28.0)	5 (45.5)	2 (14.3)
Venovenous, single-site	4 (16.0)	1 (9.1)	3 (21.4)
Venovenous, dual-site	9 (36.0)	2 (18.2)	7 (50.0)
While receiving ECMO, n (%)			
Required reconfiguration‡	5 (20.0)	0 (0.0)	5 (35.7)
Ambulation	16 (64.0)	7 (63.6)	9 (64.3)
Circuit thrombosis requiring urgent circuit exchange	1 (4.0)	1 (9.1)	0 (0.0)
Cardiac arrest	1 (4.0)	0 (0.0)	1 (7.1)
Among transplant recipients			
Bilateral lung transplant, n (%)	22 (100.0)	11 (100.0)	11 (100.0)
CAS, median (IQR)	48.3 (41.7-52.4)	45.6 (41.5-48.8)	49.2 (44.4-53.0)
Warm ischemic time, min, median (IQR)	64.0 (49.0-80.0)	78.0 (49.0-88.0)	57.0 (46.0-74.0)
Total ischemic time, min, median (IQR)	602.0 (463.0-923.5)	617.0 (506.0-830.0)	587.0 (420.0-1163.0)
Required postoperative ECMO, n (%)	4 (18.2)	2 (18.2)	2 (14.3)
Grade 3 primary graft dysfunction at 72 h, n (%)	3 (13.6)	0 (0.0)	3 (21.4)
Required postoperative tracheostomy, n (%)§	9 (42.9)	5 (45.5)	4 (40.0)
Outcomes, n (%)			
Survival to transplant‖	22 (88.0)	11 (100.0)	11 (78.6)
Survival to hospital discharge	22 (85.0)	11 (100.0)	11 (78.6)
Survival to hospital discharge in transplant recipients	22 (100.0)	11 (100.0)	11/11 (100.0)
Survival to 90 d after hospital discharge in transplant recipients¶	18 (100)	10/10 (100)	8/8 (100)
Survival to 6 mo after hospital discharge in transplant recipients#	10/10 (100.0)	6/6 (100)	4/4 (100)

*ECMO*, Extracorporeal membrane oxygenation; *BTT*, bridge to transplant; *BTD*, bridge to decision; *IQR*, interquartile range; *COPD*, chronic obstructive pulmonary disease; *CLAD*, chronic lung allograft dysfunction; *CAS*, composite allocation score.

∗ Both patients receiving mechanical ventilation prior to ECMO were extubated within 6 hours.

† All patients who received primary venoarterial ECMO underwent a “sport model” configuration^12^ via a drainage cannula placed in the right internal jugular vein and a reinfusion cannula placed through a graft sewn on the right axillary artery.

‡ Among the 5 patients who required reconfiguration after initial cannulation, 1 patient was converted from venovenous (VV) ECMO to venoarterial (VA) ECMO, 2 patients on VV ECMO received an additional VA circuit in parallel, 1 patient on VV ECMO was converted to central VA ECMO, and 1 patient was converted from dual-site VV ECMO to single-site VV ECMO. The median time to reconfiguration among patients who required reconfiguration was 2 days (IQR, 1-2 days).

§ Three 3 patients did not receive a lung transplant, and 1 patient received a tracheostomy prior to transplant.

‖ Among the patients who did not survive to transplant, 1 patient was declined for renal cell carcinoma, 1 patient was declined for clinical instability and infectious complications of diverticulitis, and 1 patient was declined for esophageal dysfunction, each discovered or developed during lung transplant evaluation.

¶ In 4 transplant recipients, 90 days have not passed since hospital discharge.

# In 12 transplant recipients, 6 months have not passed since hospital discharge.

Prior to cannulation, patients were receiving a median fraction of inspired oxygen of 1.0 (IQR, 0.95-1.0); 22 patients (80%) had a high-flow nasal cannula, 1 patient (4.0%) was receiving noninvasive positive-pressure ventilation, and 2 patients (8.0%) were receiving invasive mechanical ventilation (Table 1). Thirteen patients (52.0%) were nonambulatory at the time of cannulation.

ECMO configurations are shown in Table 1. ECMO-BTD patients tended to receive venovenous ECMO more often than venoarterial ECMO and required a greater frequency of reconfiguration. All 7 patients who received venoarterial via the “sport model”^5^ were intubated for the procedure and extubated either in the operating room following the procedure or within 6 hours of return to the intensive care unit. The sport model provides arterial inflow through the axillary artery with a cannula inserted into a graft anastomosed to the artery; drainage typically occurs through the right internal jugular vein or, less frequently, the femoral vein.^5^

Following cannulation, 24 patients (96%) were awake and free from mechanical ventilation, though 2 patients required escalation to mechanical ventilation at some point while receiving ECMO. Sixteen patients (64.0%) ambulated on ECMO, and 18 (72%) were able to be out of bed to chair. Five patients required ECMO reconfiguration (Table 1).

**Figure 1:**
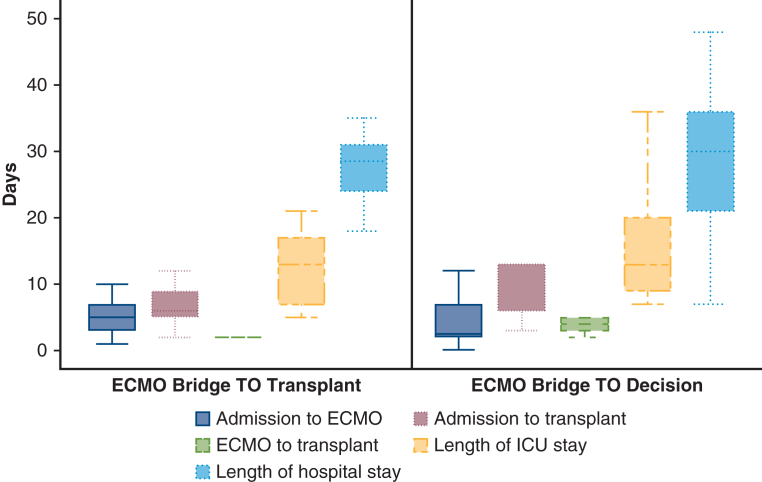
Time points from hospital admission by transplant listing status at cannulation. Extracorporeal membrane oxygenation bridge to transplant (ECMO-BTT) patients represent patients who were cannulated after being listed for transplant. ECMO bridge to decision (ECMO-BTD) patients are those who were cannulated prior to being actively listed for transplant. The lower and upper borders of the *boxes* represent the lower (25th) and upper (75th) quartiles. The *middle horizontal line* represents the median. The lower and upper whiskers represent the minimum and maximum values. Among ECMO-BTT patients, the median time from admission to ECMO was 5 days (interquartile range [IQR], 3-7 days), the median time on ECMO was 2 days (IQR, 2-2 days), the median time from admission to transplant was 6 days (IQR, 5-9 days), the median intensive care unit length of stay was 13 days (IQR, 7-17 days), and the median hospital length of stay was 28.5 days (IQR, 24-31 days). Among ECMO-BTD patients, the median time from admission to ECMO was 5 days (IQR, 3-7 days), median time on ECMO was 5 days (IQR, 4-7 days), median time from admission to transplant was 6 days (IQR, 5-9 days), median intensive care unit length of stay was 13 days (IQR, 7-17 days), and median hospital length of stay was 28.5 days (IQR, 24-31 days).

The patients were on ECMO for a median of 3.5 days (IQR, 2.0-5.0 days) before transplant (Table 1). The median time on ECMO was 2.0 days (IQR, 2.0-2.0 days) in the ECMO-BTT group and 5.0 days (IQR, 4.0-7.0 days) in the ECMO-BTD group (Figure 1). Among the whole cohort, 22 patients (88.0%) survived to lung transplant and hospital discharge. Eleven patients (100%) in the ECMO-BTT group and 11 patients (78.6%) in the ECMO-BTD group survived to transplant and hospital discharge. Among those who survived to transplant, 22 (100.0%) survived to hospital discharge, and 10 (100%) who survived for 6 months postdischarge were alive at this writing (Table 1). The ECMO-BTD patients who did not survive to transplant are described in the Table 1 footnote. Each of these deaths was related to the discovery of a preexisting medical contraindication to lung transplant during the evaluation rather than to a contraindication that developed while waiting for lung transplant. Two complications occurred on ECMO: one circuit thrombosis requiring urgent circuit exchange and one cardiac arrest during cannulation. Both patients survived to transplant and hospital discharge. No other complications, including bleeding events or systemic thromboembolism, occurred during ECMO-BTT of ECMO-BTD.

## Discussion

In this retrospective cohort study of patients who received ECMO while waiting for lung transplant, outcomes were excellent; 100% of patients in the ECMO-BTT group and nearly 80% of patients in the ECMO-BTD group received a lung transplant and survived to hospital discharge. All patients who received a transplant survived to hospital discharge and remain alive, many of whom have survived for 6 months since discharge. Importantly, time on ECMO was short, and complications while receiving ECMO were infrequent.

Our results contrast with results reported prior to the introduction of CAS showing longer time on ECMO (some exceeding 14 days),^1^ limited pretransplant survival among ECMO-BTT patients,^1^ and poor overall survival among ECMO-BTD patients.^6^ The excellent outcomes may be due to the short time on ECMO compared to historical reports, which likely reflects both allocation changes in CAS and our efforts to manage donor offers aggressively. Reducing the time on ECMO decreases the time the patient is exposed to ECMO-associated complications and likely contributes to improved outcomes. New data in the CAS era suggest less frequent use of ECMO among the pretransplant population with an overall shorter waitlist time.^4^ Although speculative, it is possible that while ECMO may be used less often in the CAS era, outcomes of patients who require ECMO may be improved.

This study has some limitations. The data are from a small sample at a single high-volume transplant institution. ECMO practices widely vary. At our institution, ECMO patient selection, timing, mode, and configuration are considered for each patient individually and based on a constellation of clinical data, including trajectory of respiratory failure, hemodynamics, transthoracic echocardiography, right heart catheterization, coronary perfusion, and anticipated differential hypoxemia. We also consider the anticipated wait time to identify donor lungs. In general, the patients who receive ECMO-BTT or ECMO-BTD are receiving 100% fraction of inspired oxygen through maximum high-low nasal cannula, noninvasive positive-pressure ventilation, or mechanical ventilation (although we preferentially cannulate prior to intubation if possible) and have limited to no ability to ambulate due to respiratory symptoms and hypoxia. Patients who received venovenous ECMO and continued to experience worsening pulmonary artery pressures and/or hemodynamics, especially in the context of rapidly progressive respiratory worsening, were transitioned to venoarterial ECMO. Our approach has been described previously.^7^ As such, results from other experiences may differ. Although no definitive conclusions about the associations between CAS and ECMO-BTT outcomes can be drawn from this study, the results suggest a promising role for ECMO in the CAS era. Further work is critical to understand which patients in this population are most likely to benefit, and whether the introduction of CAS augments this potential benefit and allows expanded provision of ECMO to higher-risk patients.

## Conflict of Interest Statement

The authors reported no conflicts of interest.

The *Journal* policy requires editors and reviewers to disclose conflicts of interest and to decline handling or reviewing manuscripts for which they may have a conflict of interest. The editors and reviewers of this article have no conflicts of interest.
